# Systematic Review of Safety of Selective Androgen Receptor Modulators in Healthy Adults: Implications for Recreational Users

**DOI:** 10.3390/jox13020017

**Published:** 2023-05-10

**Authors:** Jonathan D. Vignali, Kevin C. Pak, Holly R. Beverley, Jesse P. DeLuca, John W. Downs, Adrian T. Kress, Brett W. Sadowski, Daniel J. Selig

**Affiliations:** 1 Behavioral Biology Branch, Walter Reed Army Institute of Research, Silver Spring, MD 20910, USA; 2 Department of Gastroenterology, Naval Medical Center San Diego, San Diego, CA 92134, USA; 3 Gorgas Memorial Library, Walter Reed Army Institute of Research, Silver Spring, MD 20910, USA; 4 Clinical Pharmacology Fellowship, Walter Reed Army Institute of Research, Silver Spring, MD 20910, USA; 5 Department of Toxicology, Uniformed Services University of the Health Sciences, Bethesda, MD 20814, USA

**Keywords:** athlete, drug-induced liver injury, drug safety, recreation, selective androgen receptor modulator, tendon rupture

## Abstract

Selective Androgen Receptor Modulators (SARMs) are not FDA approved, and obtaining SARMs for personal use is illegal. Nevertheless, SARM use is increasingly popular amongst recreational athletes. Recent case reports of drug-induced liver injury (DILI) and tendon rupture raise serious concerns for the safety of recreational SARM users. On 10 November 2022 PubMed, Scopus, Web of Science, and ClinicalTrials.gov were searched for studies that reported safety data of SARMs. A multi-tiered screening approach was utilized, and any study or case report of generally healthy individuals exposed to any SARM was included. Thirty-three studies were included in the review with 15 case reports or case series and 18 clinical trials (total patients N = 2136 patients, exposed to SARM N = 1447). There were case reports of drug-induced liver injury (DILI) (N = 15), Achilles tendon rupture (N = 1), rhabdomyolysis (N = 1), and mild reversible liver enzyme elevation (N = 1). Elevated alanine aminotransferase (ALT) was commonly reported in clinical trials in patients exposed to SARM (mean 7.1% across trials). Two individuals exposed to GSK2881078 in a clinical trial were reported to have rhabdomyolysis. Recreational SARM use should be strongly discouraged, and the risks of DILI, rhabdomyolysis, and tendon rupture should be emphasized. However, despite warnings, if a patient refuses to discontinue SARM use, ALT monitoring or dose reduction may improve early detection and prevention of DILI.

## 1. Introduction

Selective Androgen Receptor Modulators (SARMs) are non-steroidal compounds with favorable oral bioavailability that were developed in the early 2000s in an attempt to overcome the pharmacologic and pharmacokinetic limitations of steroidal androgen receptor agonists (i.e., testosterone and DHT), which have known associations with liver and heart disease [1]. SARMs have been trialed as a pharmacologic intervention to improve a wide variety of conditions such as cancer-associated morbidity, deconditioning after hip fracture, stress incontinence, and benign prostatic hyperplasia [2]. Solomon et al. provided a comprehensive review of current clinical applications [3]. Despite a strong warning from the Food and Drug Administration (FDA) [4], SARM abuse is increasingly popular amongst recreational and professional athletes as a perceived means to improve performance [5]. The prevalence of SARM abuse is uncertain; however, estimates of the global lifetime prevalence rate for use of anabolic-androgenic steroids are 3.3%, with a prevalence rate of 6.4% in males and 1.6% in females [6]. According to a British Army survey of 3168 soldiers in training, 1.1% reported use of anabolic steroids, 2.0% reported use of growth hormone, and 4.2% reported use of other anabolic-androgenic agents, with a strong association in young soldiers [7]. According to a Department of Defense (DoD) Health-Related Behaviors Survey in 2015, 4.1% of respondents reported use of anabolic steroids at least one or more times in their life, with over 20% of steroid users reporting obtaining a prescription outside of the military health system [8]. In the professional athletic setting, the World Anti-Doping Agency (WADA) banned SARMs. However, despite the ban there was a rise in positive tests for SARMs between 2015 and 2019 [9]. More recent social media trends have observed a notable increase in searches and views regarding topics related to SARMs, particularly on platforms such as TikTok, Reddit, and YouTube that include a relatively large portion of adolescent and young adult populations [10].

In the years 2020–2022, there has been a rapid increase in the number of published case reports of drug-induced liver injury (DILI) associated with SARM abuse [11,12,13]. There have also been case reports of rhabdomyolysis and tendon rupture associated with SARM abuse [14,15]. The mechanisms for these injuries remain unknown. However, Koller et al. postulated that systemic accumulation of a multitude of metabolites with repeat SARM dosing likely contribute to the development of a secondary immune response targeting the liver [16]. Machek et al. provided a more comprehensive review on the possible mechanisms of harmful effects of SARMs [17]. The growing popularity of SARMs, possibly due to social media influence, rapid increase in reported serious adverse events associated with SARM abuse, and general interest in performance-enhancing drugs amongst recreational and professional athletes represents an emerging public health concern. Several of the DILI case reports provide important insight into possible risk factors and mechanisms of DILI, such as significantly increased doses taken compared with those taken in clinical trials and a large number of SARM metabolites which may have deleterious effects [11,16,18]. However, to our knowledge, there is currently no systematic review of the safety of SARMs in healthy populations that reasonably extrapolate to the population of SARM abusers. Therefore, we performed this systematic review of the safety of SARMs in healthy populations to better describe the characteristics of SARM-associated DILI and compare safety data from case reports with the clinical trials. This assists in generating safety signals and clinical strategies to improve safety of SARM abusers and individuals considering SARM abuse.

## 2. Materials and Methods

### 2.1. Inclusion Criteria

The focus of this review is the implications of using SARMs for performance enhancement in highly active and military populations. Therefore, any case report or study was included if it provided sufficient clinical safety data and the patient population was generally healthy. Of note Padappayil et al. reported a case of myocarditis associated with SARM use [19]. However, this case report was excluded due to confounding comorbidities in the patient such as type 1 diabetes mellitus and substance use disorder requiring maintenance buprenorphine therapy. The rationale for exclusion is further elaborated in the discussion. Patient populations with cancer or chronic diseases were excluded as the risk-to-benefit of SARM therapy changes greatly in those populations compared with healthy individuals. Some chronic diseases present a wide spectrum of illness that studies may have included or excluded depending on the disease severity of the population. For example, Mohan et al. was excluded because the patient population studied had baseline severe chronic obstructive lung disease (COPD) [20]. Papanicolaou et al. was included because sarcopenia is an age-related loss of muscle mass, and the authors were careful to exclude patients with any chronic disease or cancer [21,22]. Nash et al. performed a retrospective study finding 22 cases of DILI associated with androgenic-anabolic steroid (AAS), SARMs, and other body building supplements [23]. The dataset in this study included 10 cases of SARM-associated DILI. This study provided significant insight into AAS and SARM-related DILI; however, it was not clear if the patients were exclusively taking SARMs or were taking multiple compounds of different drug classes. Therefore, this study was excluded. No age limit was applied to the inclusion criteria as individuals of all ages may enjoy being active and improving muscle strength and physical appearance.

### 2.2. Search Strategy

On 10 November 2022, PubMed, Scopus, Web of Science, and ClinicalTrials.gov were searched for studies that reported safety data of SARMs in healthy individuals. This review was registered in PROSPERO (CRD42022380525) and the PRISMA 2020 checklist was used as a guide to perform, complete, and report the review. The search string utilized was “(SARMs[Title/Abstract] OR Selective Androgen Receptor Modulators[Title/Abstract] OR Ostarine OR Enobosarm OR GTX-024 OR MK2866 OR Andarine OR Ligandrol OR LGD-4033 OR VK5211 OR LY2452473 OR TT-701 OR TT701 OR Testolone OR RAD140)”. A total of 1225 titles were found across the databases (715 after duplicates removed) and an additional 33 titles were found in ClinicalTrials.gov. Two investigators (DJS and JWD) independently screened the titles and/or abstracts. Following this stage of review, 100 articles were identified that may have met inclusion criteria, of which 74 were retrieved. Two investigators (JPD and ATK) independently screened the 74 full articles for inclusion and disagreements were resolved by a single author (DJS) resulting in 29 studies included from the databases. Clinicaltrials.gov was screened sequentially after the screening and inclusion of studies from PubMed, Scopus, and Web of Science was complete. From Clinicaltrials.gov, 13 studies in cancer populations, 4 studies with no SARM, 2 studies terminated with no results (extension studies stopped after lack of efficacy found for Gx-024 as a therapy for stress incontinence), 7 duplicate studies found in the other databases, and 3 studies that met the inclusion criteria but had no results (NCT01538420, NCT01275157, NCT03264651) were excluded. The PRISMA 2020 flow diagram summarizing the search results is presented in the Supplementary Materials.

### 2.3. Data Collection, Analysis, and Safety Outcomes of Interest

All data were plotted and analyzed using R (version 4.2, R Foundation for Statistical Computing, Vienna, Austria) and R Studio (version 2022.07.2 + 576, RStudio Team, Boston, MA, USA). Data were collected by a single author (DJS) and reviewed by KCP. Studies were categorized into case reports and clinical studies and are summarized in Table 1 and Table 2. Data extracted from all studies, when available, were author, publication year, study design, study population, number of subjects/patients, number of subjects/patients exposed to SARM, the specific SARM, dose and schedule of SARM, gender age, weight, and alcohol use.

Many case reports described the change in liver enzymes over time, which was either manually tabulated or digitized using WebPlotDigitizer (Version 4.6, Ankit Rohatgi, Pacifica, CA, USA) depending on the presentation of data in the case report. Additionally, for case reports, the R-factor (or R-ratio or R-value) was extracted as reported in the case (Table 1) [49]. The R-factor is mathematically defined as:

(1) $$\frac{\left(\frac{ALT}{{ULN}_{ALT}}\right)}{\left(\frac{ALP}{{ULN}_{ALP}}\right)}$$

where ALT is the alanine aminotransferase, ULN_ALT_ is the upper limit of normal for alanine aminotransferase, ALP is the alkaline phosphatase, and ULN_ALP_ = is the upper limit of normal for alkaline phosphatase [50]. The R-factor additionally was calculated at each available time point using Equation (1) above to evaluate trends in the R-factor over time. As most case reports did not disclose the upper limit of normal (ULN), when calculating R-factors the ULN were assumed to be 40 I/U and 147 IU/L for alanine aminotransferase (ALT) and alkaline phosphatase (ALP), respectively [51,52].

For the clinical trials, the proportion of patients with ALT elevation greater than 2 times the ULN was extracted. To explore the possibility of a dose–response relationship between SARM use and ALT elevation, doses were categorized as low, medium, or high. For each respective SARM, doses were considered low if they were in the 1st quartile, medium if in the 2–3rd quartile, and high if in the 4th quartile of doses across all included trials of the respective SARM. Otherwise, safety was described qualitatively. Given all safety outcomes were of interest, adverse events may be rare and not discovered in registration trials, and there was no attempt to estimate a measure of effect, a formal risk of bias assessment was not performed. Limitations of specific studies are considered in the discussion section.

Missing data were excluded from the analysis. In cases where a demographic variable was reported in a clinical trial as a range, the mean of the range was assumed to be the observed mean of the demographic variable of interest. Some SARMs have multiple names. For pooled analysis, all formulations of Enobosarm (Ostarine, MK-2866) were labeled as Enobosarm.

## 3. Results

### 3.1. Summary of Case Reports

There were 15 unique case report manuscripts with a total of 18 cases (Flores et al., Koller et al. and Perananthan et al. each reported two cases in a single manuscript) [16,26,30]. The large majority (88%, N = 15) reported DILI associated with SARM use. Other significant adverse events included one case of severe rhabdomyolysis (Kintz et al.) and one case of bilateral asynchronous Achilles tendon ruptures (Gould et al.) [14,15]. The case of rhabdomyolysis occurred in a 43-year-old male after cycling approximately 75 miles. Ten days prior to hospital presentation, the patient had taken a single dose of 20 mg MK-2866 and a 4-day course of 20 mg GW-1516 daily. The case of Achilles tendon rupture occurred in a 36-year-old male competitive powerlifter. The initial tendon rupture occurred in the right Achilles while playing dodgeball. He underwent surgical correction and 4-days post-operatively experienced a left Achilles tendon rupture while hopping on his left leg. Approximately 5 weeks prior to initial injury the patient completed two 4-week cycles of SARM compounds (cycle 1: ostarine alone, cycle 2: ostarine and cardarine). There was also a report of an experimental efficacy trial in a single 25-year-old male taking 10 mg LGD-4033 daily and 15 mg MK-677 daily (Cardaci et al.) [27]. There were no significant adverse events in this report; however, there was a reversible increase in ALT from 20 to 61 IU/L and reversible decrease in high-density lipoprotein (HDL) from 55 to 35 mg/dL after 5 weeks of SARM use.

In the remaining 15 DILI cases, patients were 100% male with a mean age of 33.5 ± 9.3 years. Weight and race were not consistently reported. Alcohol use was also inconsistently quantified and reported. Two cases reported a significant history of alcohol use without quantifying [18,26], one study reported that the patient denied alcohol use [12], two studies reported and quantified less than weekly alcohol use [13,31], one study reported insignificant alcohol use without quantifying [16], and the remaining seven cases did not report alcohol use, but implied alcohol use was not significant [11,16,24,25,26,28,30,31]. Use of concomitant medications including acetaminophen were rarely reported and only Lee et al. reported acetaminophen levels, which were within normal limits [29].

Patients were reported to commonly use SARMs for muscle building and described as having interest in being athletic and/or a bodybuilder. Two patients were noted to be active-duty military [25,29].

The specific SARM was identified in all but one case (Khan et al.) [30]. Of cases reporting a specific SARM, the most common SARM used overall was LGD-4033 (57.1%, N = 8), followed by RAD-140 (42.9%, N = 6), Enobosarm (21.4%, N =3), and a single case of YK11 use (Lee et al.) [29]. The majority of cases reported single SARM use (78.6% of cases, N = 11). Khan et al. reported use of a SARM supplement and it is unclear if this was one or multiple SARMs [28]. Of the case reports with single SARM use, the most common SARM was LGD-4033 (45.5%, N = 5), followed by RAD-140 (36.4%, N = 4) and Enobosarm (18.2%, N = 2). Two cases reported the use of two SARMs (Barbara et al., Koller et al.) [16,18] and one report involved three different SARMs (Lee et al.) [29]. The total daily dose was reported in only four cases with mean 12.6 ± 11.1 mg. The frequency was reported in five cases and SARMs were most commonly taken daily (80% N = 4), while one case reported dosing twice daily. The time course of initiation of SARM to symptom onset was unclear; however, most cases reported the total duration of SARM consumption prior to presentation (mean 6.7 ± 3.3 weeks) and reported the time from discontinuation to initial symptoms ranged from 0 to 60 days. Of the eight studies that reported time from discontinuation to initial symptoms the mean was 14.1 ± 20 days.

**Figure 1:**
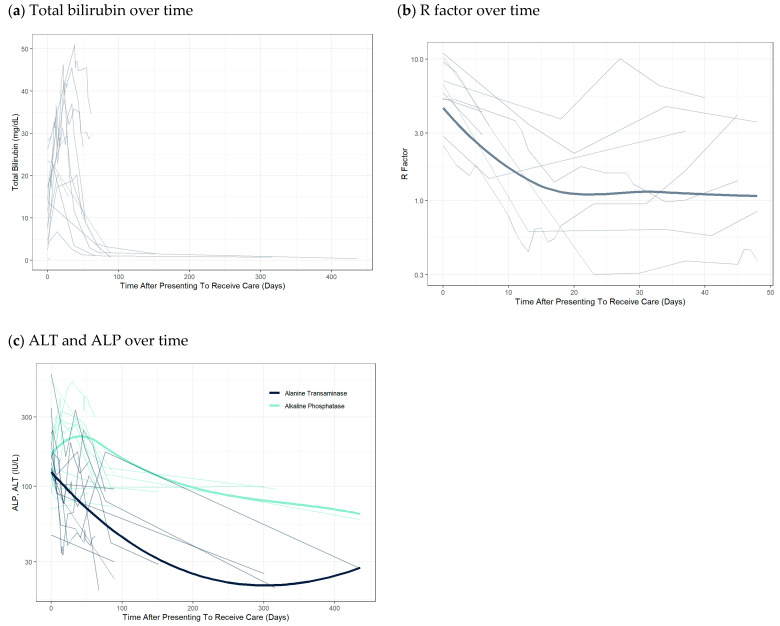
Trend of alanine aminotransferase (ALT), alkaline phosphatase (ALP), total bilirubin, and R-factor over time from case reports. Thick lines are locally estimated scatterplot smoothing (LOESS) trend lines and thin lines represent individual patient data.

The majority of the patients described in the case reports presented with symptoms of hyperbilirubinemia after exposure to SARMs. The most commonly reported symptoms were jaundice and/or dark-colored urine (93.3%, N = 14). Weight loss and fatigue were also noted in some cases, with one case reporting a 40-pound weight loss [11]. The marked elevations in bilirubin levels relatively early on can be observed (Figure 1a). Initially, the pattern of liver injury appeared to favor a hepatocellular pattern. However, the R Factor decreased over time, suggesting a prevailing cholestatic pattern of injury (Figure 1b). Liver biopsies were obtained in 66.6% (N = 10) case reports. The biopsy results were universally supportive of cholestatic injury. Predominant findings on liver biopsy were cholestasis which is typically seen in cholestatic DILI from anabolic drugs and oral contraceptives [53]. There were thirteen cases with reported imaging findings. Eight of these patients had some form of cross-sectional imaging with either a CT or MRI/MRCP. The majority of the imaging findings were normal with one patient having hepatic steatosis detected by ultrasound, and two patients with either hepatomegaly or splenomegaly [11,13,25].

Generally, patients recovered completely by 3–6 months. Some patients took 10 months to 1 year for labs to normalize [13,26]. Total bilirubin and ALP peaked around days 25–50, with median time to peak at 18 and 12 days from presenting to receiving care, respectively. In contrast, ALT peaked early and generally down-trended thereafter (Figure 1c). After 50 days from presentation to receipt of care, almost all patients had downtrends in all liver-associated enzyme tests. (Figure 1a,c) Mean peak values for total bilirubin, ALP, and ALT were 28.5 ± 13.4 mg/dL, 283.1 ± 160.9 IU/L, and 226.3 ± 142 IU/L, respectively. All therapy was supportive, and the most commonly prescribed treatment was ursodeoxycholic acid (46.7%, N = 7). Cholestyramine was co-prescribed in four of those cases. One case reported a trial of 300 mg N-acetyl cysteine intravenously four times daily while hospitalized [16].

### 3.2. Summary of Clinical Studies

#### 3.2.1. General Characteristics of Studies

Table 2 summarized the study characteristics. There were 18 clinical studies describing safety, efficacy, or pharmacokinetics in subjects taking SARMs. One study was a cross-sectional survey of SARM users that inquired about self-prescription patterns and perceived positive and negative effects [46]. The remaining 17 were clinical trials. Nine of the trials were early phase tolerability, pharmacokinetic, or dose finding studies [32,33,35,36,37,39,40,42,47], two trials were bioavailability or drug-interaction studies [38,43], one was a small pilot efficacy study [41], and the remainder were larger efficacy trials [21,34,44,45,48]. The 17 clinical trials tested 13 unique SARM compounds and included a total of 2136 patients with 1447 patients exposed to a SARM. The most commonly trialed SARM was Enobosarm (N = 5 trials), followed by LGD-4033 (N = 3 trials), LY2452473 (N = 3), OPK-88004, and GSK2881078 (N = 2 trials), with the remaining compounds each being tested in one clinical trial. The patient populations were generally healthy, although SARMs were investigated for different indications such as gain of muscle mass/function, stress urinary incontinence, erectile dysfunction, and symptomatic benign prostatic hyperplasia. The median and range of mean ages across all trials was 57 (24–77) years (13 trials reported age) and similarly median weight and range was 81.2 (51.9–88.5) kg (7 trials reported weight). Nine studies included only men (N = 948, exposed N = 648) [33,35,36,37,38,43,44,47,48], four studies included only females (N = 768, exposed N = 492) [21,32,41,45], and the remaining four studies included both males and females. Of these studies, Clark et al. included 24.2% females (N = 24, exposed N = 18) [39], Dalton et al. included 50% females (N = 60, exposed N = 48) [34], Neil et al. included 50% females (N = 46, exposed N = 31) [40], and Ristic et al. included 76.9% females (N = 83, exposed N = 62) [42]. Efimenko et al. reported 343 survey responders that were most commonly adult healthy men (98.5%) with 72.3% of survey respondents between the ages of 18–29 years old [46].

#### 3.2.2. Serious Adverse Events

Serious adverse events (SAEs) were reported in six of the clinical trials. Clark et al. (GSK2881078) reported one subject, who developed chest pain, had an emergent cardiac catheterization which was negative and was followed but withdrawn from further SARM therapy [39]. Drug association was not made clear in the manuscript for this SAE. Another patient developed a maculopapular rash and ALT elevation 2.9 times the ULN consistent with a drug reaction. Two other subjects in the same study developed rhabdomyolysis after strenuous physical activity but these SAEs were not considered drug-related. Papanicolaou et al. (MK-0773) reported 27 SAEs occurred in 21 subjects, and eight were drug-related [21]. Five of the eight drug-related SAEs were attributed to elevations in ALT and AST and these five subjects were withdrawn from the study. Reassuringly, all subjects’ ALT and AST levels normalized after discontinuation of the SARM. The remaining drug-related SAEs were not clearly reported by Papanicolaou et al. Pencina et al. (OPK-88004) reported three SAEs, which were not considered to be drug-related [21,47]. One subject underwent coronary artery bypass grafting, one subject was diagnosed with lung and liver cancers, and one subject in the placebo group was diagnosed with renal cancer. In NCT03241342 (GTx-024), there were five SAEs reported, including acute myocardial infarction, goiter, hip fracture, cerebrovascular accident, and dysesthesia, but the association to drug was not reported [45]. In NCT01160289 (LY2452473), SAEs occurred in four subjects randomized to LY2452473 [44]. These SAEs included lobar pneumonia, humerus fracture, tubulointerstitial nephritis, pulmonary embolism, arrhythmia, and cardiac arrest; however, drug causality was not reported. In NCT03297398 (OPK-88004), SAEs occurred in two subjects including coronary artery disease, bile duct obstruction, and cholangitis [48].

#### 3.2.3. Hepatobiliary Adverse Events

**Figure 2:**
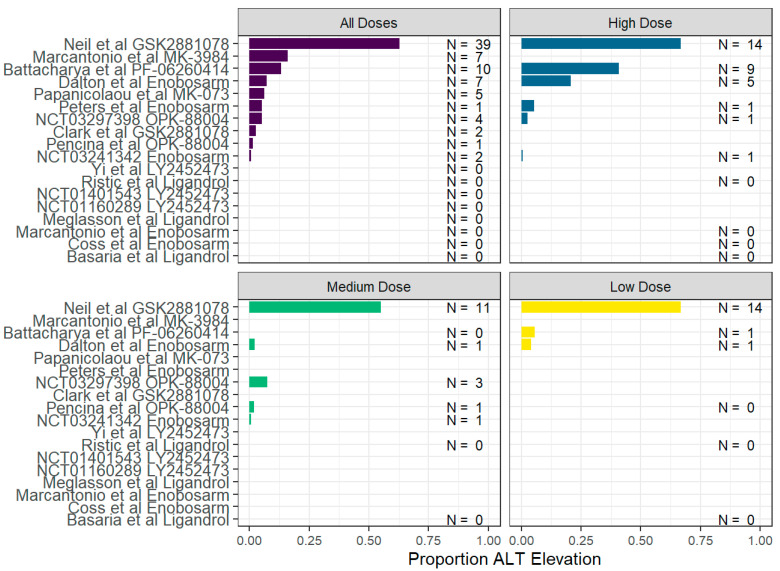
Proportion of alanine aminotransferase (ALT) elevation observed in clinical or observational trials [21,32,33,34,35,36,37,38,39,40,41,42,43,45,47,48]. For each respective SARM, doses were considered low if they were in the 1st quartile, medium if in the 2–3rd quartile, and high if in the 4th quartile of doses across all included trials of the respective SARM.

Elevations in liver enzymes greater than 1 to 2 times the ULN were reported in 10 clinical trials. Mean and median proportion of subjects experiencing LAE elevation across all trials were 7.1% (N = 78) and 1.3%, respectively, with a range of (0–62.9%). There was large variability observed even between two separate trials of the same compound. For example, Clark et al. and Neil et al. (GSK2881078) reported 4.3% and 62.9% subjects with ALT elevation, respectively [39,40]. Similarly, for Enobosarm, Dalton et al. and Peters et al. reported ALT elevations in 7.3% (N = 39) and 2.6% (N = 2) of subjects, respectively [34,41]. However, Coss et al. and Marcantonio et al. reported 0% of subjects experiencing ALT elevation and NCT03241342 reported only 0.6% (N = 2) [32,38]. Importantly, Coss et al. was not a repeat dose study and doses of SARMs may have differed between trials, which may explain some of the variability [38]. Figure 2 summarizes the proportion of subjects in each trial with elevated ALT and suggests that the majority of cases were associated with higher doses of SARM. Overall, GSK2881078 was associated with the highest rates of ALT elevations; however, this finding was largely driven by Neil et al. (Figure 2) [40]. Most patients only had mild ALT elevations and were able to continue the SARM with ALT returning to baseline by day 28 of therapy.

Similar to GSK2881078, most ALT elevations for other compounds were mild. However, there were several subjects that had greater than four times the ULN elevations in ALT (2 in Neil et al., 1 in Dalton et al., and 1 in Bhattacharya et al. [34,37,40]) with the greatest reported ALT at 343 IU/L in a patient receiving PF-06260414 [35]. Other hepatobiliary enzymes were not as consistently reported. Elevation in alkaline phosphatase (ALP) was only reported in one patient receiving GTX-024 in the clinical trial NCT03241342 [45]. Elevation in serum bilirubin levels were not reported in any of the clinical trials, although bile duct obstruction and cholangitis were two SAEs reported in NCT03297398 observing OPK-88004 [48]. In addition to the NCT03241342 trial, two other trials analyzed the ALP levels and bilirubin levels. Both Pencina et al. (OPK-88004) and Neil et al. (GSK2881078) reported a decrease in ALP and unchanged bilirubin levels [40,47]. Pencina et al. separately reported bone-specific ALP which also demonstrated a decrease. Based on the overall findings, the liver injuries observed were mostly mild hepatocellular injuries [47]. SARMs associated with liver injury from the clinical trials were PF-06260414, GSK2881078, GTX-024, MK-3984, MK-0773, and OPK-88004.

#### 3.2.4. Other Non-Serious Adverse Events

Eight clinical trials reported reductions in HDL. The SARMs in the studies that reported a reduction in HDL were LGD-4033, PF-06260414, GSK2881078, OPK-88004, and LY2452473 [36,37,39,40,44,47]. A decrease in total testosterone levels in male patients was also observed in the studies that reported hormonal data. The SARMs that were used by these patients were LGD-4033, RAD-140, and MK-2866. In four studies that reported hormonal levels, in addition to reduced total testosterone levels, there were reduced levels of sex hormone-binding globulin (SHBG). Commonly reported symptoms and findings for all SARMs were headaches, dry mouth, and upper respiratory infections (URIs), constipation, dyspepsia, and nausea. Papanicolaou et al. reported elevated hemoglobin and hematocrit greater than 3% in approximately 5% of patients [21]. There was also a trend towards an increase in systolic blood pressure with the SARM group having a mean increase of approximately 3 mmHg from baseline, compared with placebo, which had a decrease of approximately 2 mmHg from baseline. In contrast, Pencina et al. (OPK-88004) found inconsistent small elevations of hematocrit (<1%) in only one subgroup (5 mg); however, hematocrit decreased in the 15 mg subgroup and remained unchanged in the 1 mg subgroup [47]. NCT03241342 (GTX-024) did not specifically report hemoglobin/hematocrit levels but reported one event of polycythemia and one event of thrombocytosis. Hypertension was reported in nine subjects receiving GTX-024 in NCT03241342; however, drug causality was not mentioned [45]. The clinical trials did not commonly investigate or report testicular size, development of acne, or mood swings. Of note, these were the most common adverse effects of survey respondents in the study by Efimenko et al., with reported rates of 22.4% for mood swings, 20.7% for decreased testes size, and 15.2% for acne [46]. Elevations in blood pressure were also noted in Efimenko et al., but not quantified.

## 4. Discussion

Multiple reports of serious and potentially life or limb threatening adverse events have been reported in the last 2–3 years. SARM use has become increasingly popular among younger males in particular, possibly as a result of the growing popularity of a large number of social media fitness influencers [54]. We systematically reviewed the literature and concisely presented the most important safety findings associated with SARMs. This study elucidates trends in SARM-associated DILI, raises awareness of other possible lesser-known significant adverse events associated with SARM use, and allows for comparison of the adverse events found in clinical trials to those found in case reports.

The clinical trials clearly demonstrated a signal for potential of DILI. Although the mechanism is not clear, Neil et al. evaluated microRNA-122 levels and found an association between SARM use and mild hepatocellular liver injury [40]. Koller et al. discussed at length the theoretical mechanisms for DILI and hypothesized SARM-associated DILI is idiosyncratic, with elevated doses playing a significant role [16]. This hypothesis is consistent with the strong suggestion of dose response of SARM use to a proportion of subjects with ALT elevation in clinical trials. Fortunately, in the closely monitored clinical trial setting, subjects were either withdrawn from SARM therapy or were closely followed, and ALT generally quickly returned to baseline. In contrast, patients in case reports generally reported taking SARMs at 4–10 times the doses in clinical trials and were commonly using multiple SARMs. Furthermore, they were not observed with serial ALT monitoring, and only presented for clinical care with significant symptoms of biliary obstruction. Of note, many patients in the case reports were reported to initially have a hepatocellular pattern of DILI. Over time, however, the DILI consistently converted to a cholestatic pattern (Figure 1b). This finding is again consistent with the clinical trials, where the majority of patients had hepatocellular injury and were able to recover with early withdrawal of SARM. Consequently, although clinicians should strongly discourage the use of SARM supplementation, if a patient continues to abuse SARMs despite warnings from the clinician, serial ALT monitoring and encouraging lower doses are two strategies that may reduce the risk of DILI and improve patient safety. It is important to inform the patient that these strategies do not endorse SARM use and are only being used as a last resort measure to ensure the safety of the patient while maintaining a confidential patient–provider relationship.

Aside from DILI and elevated ALT, there were concerning safety signals for musculoskeletal side effects. Both Clark et al. (GSK2881078) and Kintz et al. (case report) reported rhabdomyolysis [15,39]. In both the case report and Clark et al., the subjects were reported to have performed significant physical activity, and the association with the drug is not certain [39]. Of note, there have been several case reports relating anabolic steroid use to the development of rhabdomyolysis [55,56,57,58]. The mechanism underlying proposed anabolic steroid-induced rhabdomyolysis is unknown, and the causality is not fully established. However, SARMs have many metabolites and high potential for off-target effects [35,59,60,61,62]. Furthermore, it is rational to hypothesize that individuals starting a workout supplement may perform more strenuous activity if the individual believes that the supplement will enhance performance. Therefore, individuals choosing to take SARMs should be aware of the possibility of rhabdomyolysis, and only gradually increase exercise activity over time. Furthermore, it would be reasonable to screen for SARM use when patients present rhabdomyolysis.

A striking case report was reported by Gould et al., asynchronous bilateral tendon ruptures [14]. Of note, androgenic anabolic steroids have been linked to tendon rupture through case reports and a cross-sectional survey study [14,63,64]. However, the mechanical and biological effects of anabolic steroids on tendons and causality of tendon rupture remain unclear [65]. Nevertheless, similar to rhabdomyolysis, given the multiple metabolic pathways of SARMs and potential for off-target effects, in combination with reports of tendon rupture associated with SARMs and anabolic steroids, there is significant concern for tendon injury in SARM users. Active SARM users should be made aware of the association of tendon rupture, and patients presenting with ruptured tendons should be screened for SARM use.

Regarding cardiovascular health, dose-dependent, reversible reductions in HDL were observed in many of the clinical trials, regardless of the individual SARM. Papanicolaou et al. reported mild elevations in hemoglobin/hematocrit and a trend for increase in systolic blood pressure [21]. Furthermore, although unclear if related to SARM therapy, NCT03297398 reported a subject in the SARM group to have coronary artery disease and in NCT01160289 one subject on SARM therapy was reported to have a pulmonary embolus [44,48]. The convergence of decreasing HDL, increasing hemoglobin, and increasing blood pressure in combination is highly concerning. Especially since there is a likely causal association of anabolic steroid use and adverse cardiovascular outcomes [66]. Therefore, although patients should be strongly discouraged to initiate or continue SARM supplementation, patients that use SARMs should be screened and monitored for underlying cardiovascular disease throughout the duration of their use. Further, SARM users should be educated about the possible adverse cardiovascular outcomes.

There was not enough evidence to quantify risk of use of concomitant medications, in particular acetaminophen, on developing SARM-associated DILI. However, cases were generally inconsistent with acetaminophen toxicity as peak aminotransferases were generally less than 1000 I/U and liver biopsy results did not demonstrate zone 3 hepatic necrosis [67,68]. Nevertheless, this does not rule out acetaminophen as a risk for SARM-associated DILI or vice versa. SARMS have been found to have a multitude of metabolites, including phase II metabolites through sulfation and glucuronidation. Acetaminophen liver injury occurs when a higher percentage of acetaminophen is metabolized via the cytochrome P-450 pathway resulting in a build-up of toxic metabolites [67]. Therefore, it is reasonable to hypothesize that concomitant SARM and acetaminophen use may place an individual at higher risk for acetaminophen-induced liver injury if doses of SARM and acetaminophen are high enough to overwhelm the phase II metabolism pathway.

Limitations to this review include the inability to determine causality of SARM therapy to the reported adverse event and insufficient data to determine risk in subgroups. For example, Padappayil et al. reported a case of myocarditis associated with RAD-140 use [19]. However, the patient had a history of type 1 diabetes mellitus with poor glycemic control (HbA1C 10.2 mmol/mol) and a history of substance abuse requiring buprenorphine maintenance therapy. Diabetes mellitus in general is a risk factor for cardiomyopathy, in particular type 1 diabetes mellitus with poor glycemic control is a risk for cardiac autoimmunity [69,70]. Furthermore, viral infections are the most commonly reported cause of myocarditis in the United States [71]. Although Padappayil et al. reported a negative upper respiratory viral panel, this does not definitively rule out a viral infection as the cause of myocarditis [72]. This case highlights the importance of using probability scales such as the Adverse Drug Reaction Scale to better standardize the reporting of causality [73]. Nevertheless, although causality was not able to be determined definitively in many cases presented in this review, one main purpose of this review was to highlight all possible significant adverse effects related to SARMs. This is of particular importance given adverse events may be underreported in a clinical trial setting, further highlighting the need for continued pharmacovigilance after trial completion [74]. Finding three cases of rhabdomyolysis across the case reports and clinical trials is one example of how this review may raise awareness for new safety signals other than DILI. Regarding DILI, the rates of ALT elevation were not consistently reported by subgroups of age, gender, or weight. Therefore, inference was not able to be made regarding these subgroups. All patients in case reports were healthy males. This does not imply females are at lower risk for DILI. Rather, males appear to be far more likely to use SARM supplementation, as evidenced by Efimenko et al., where nearly 100% of survey respondents using SARMs were male [46]. Therefore, females considering SARM supplementation or actively using SARM supplementation should be counseled and monitored to the same standard as males. Alcohol use and precise quantification were not consistently reported in the case reports. Only two cases reported a significant history of alcohol use and therefore alcohol as a risk factor for SARM-related DILI remains unclear.

## 5. Conclusions

SARM use may be associated with DILI, rhabdomyolysis, tendon rupture, and adverse cardiovascular outcomes. Despite clear and repeated warnings by the FDA regarding use of these unapproved drug products, they continue to be available online and used in the fitness and athletic communities. Providers and public health officials should strongly discourage SARM supplementation and strongly counsel patients on the potential risks of SARM use. SARM-related DILI appears to be dose-related and may initially present with a hepatocellular injury, later converting to a mixed or cholestatic injury. If a patient continues to use SARMs despite warnings, ALT monitoring and dose reduction are strongly recommended in order to detect and reduce the risk of potential DILI as early as possible. The clinician should make clear to the patient that ALT monitoring and dose negotiations do not endorse SARM use. Rather, these are only last resort methods to ensure safety while maintaining a confidential and trusted patient–provider relationship.

## Figures and Tables

**Table 1:** Summary of case reports.

Study	Year	Age (Years)	SARM	Dose (mg)	Duration (Weeks)	Time from Stopping to Symptoms (days)	Presenting Symptoms	Imaging	Biopsy	R Factor	Treatment and Outcome
Baliss et al. [24]	2020	31	RAD-140		12		3 days of epigastric pain, jaundice and pruritis, scleral icterus	MRI abdomen normal			Labs down-trended after 1 month
Barbara et al. [11]	2020	32	LGD-4033	10	2	1–7	diffuse itching, jaundice, acholic stool, intermittent abdominal pain, nausea, 40 lbs. weight loss, scleral icterus	US and CT-hepatomegaly MRCP, small hepatic cyst and splenomegaly, otherwise normal	Cholestatic hepatitis with mild portal, periportal, perisinusoidal fibrosis		
Barbara et al. [18]	2020	52	RAD-140, LGD-4033	17.86, 10	7	1–7	Right upper quadrant pain, pruritus, and greasy diarrhea, scleral icterus, jaundice	MRI abdomen normal	Diffuse centrilobular canalicular cholestasis, mild portal and periportal fibrosis	0.2	Stop alcohol consumption, improvement in labs at 3 months
Bittner et al. [25]	2020	34	LGD-4033	7.5	4	7	Decreased appetite, worsening pruritus, dark amber urine, and cognitive ‘clouding’, jaundice, scleral icterus	US normal. CT abdomen–mild splenomegaly	Inflammation and diffuse cholestasis		
Flores et al. [26]	2020	24	LGD-4033		9	7	jaundice, anorexia, nausea, lethargy, 5 kg weight loss	US normal		8.22	Labs normalized after 4 months
Flores et al. [26]	2020	49	RAD-140		4	20–30	Jaundice and itching		Moderate cholestasis with ductopenia and minimal fibrosis and inflammation,	5	Ursodiol and cholestyramine, labs normalized after 12 months
Bedi et al. [12]	2021	40	Enobosarm		8		Jaundice, anorexia, weight loss, lethargy, and diarrhea, scleral icterus	US, CT abdomen, and MRCP normal	centrilobular cholestasis with yellow-green bile in hepatocytes and canaliculi	0.8	Improvement in labs over several months of follow up
Gould et al. [14]	2021	36	Ostarine and Carderine		8	35	Asynchronous bilateral Achilles tendon rupture after two 4-week cycles of SARM compounds				
Kintz et al. [9]	2021	43	MK-2866, Carderine	20, 20	0.7	10	Patient presented with severe rhabdomyolysis after cycling 74.6 miles with extreme elevations of ALT, AST, and CK				
Koller et al. [16]	2021	19	LGD-4033		7	0	Patient cycled 4 weeks on, 4 weeks off. Restarted the cycle for 3 weeks and stopped after noticing dark urine, yellow sclera, and thinner light-colored stools	US abdomen-normal	Mild septal fibrosis, canalicular cholestasis in the hepatocytes with numerous biliary plugs	3.9	1000 mg ursodeoxycholic acid (UDCA) daily for 2 mo. Labs normalized after 3 months
Koller et al. [16]	2021	28	LGD-4033, Ostarine			0	For 3 months took unknown amount of SARM. After 3-week break, a formulation of SARM bought on internet was taken for 4 doses, reasons for seeking medical care unclear	MRI abdomen hepatomegaly without biliary pathology	Mild bridging fibrosis, destruction of bile ducts, centrilobular canalicular cholestasis with numerous bile plugs	3.3	300 mg intravenous N-acetyl cysteine 4 times daily, 1000 mg oral UDCA daily, and 450 mg silymarin daily. Labs significantly improved at 3 months
Cardaci et al. [27]	2022	25	LGD-4033, MK-677	10, 15	5		This was an efficacy case report, no SAE’s reported, however, ALT increased from 20 IU/L to 61 IU/L after 5 weeks. The ALT returned to baseline after 4 weeks off SARM				
Khan et al. [28]	2022	29			4		Jaundice, pruritus, fatigue, scleral icterus, light-colored stools, dark urine	CT abdomen-normal	Centrilobular bile stasis with lipofuscin pigment along with collection of neutrophils within lobular parenchyma		Labs normalized by 6 months
Lee et al. [29]	2022	23	LGD-4033, RAD-140, YK11		12	7	Jaundice, scleral icterus, decrease in appetite, and worsening pruritis	MRI and MRCP normal		0.8	Ursodeoxycholic acid and hydroxyzine, labs normalized within a year
Peranathan et al. [30]	2022	30	RAD-140				Jaundice and fatigue	Unspecified image normal	Acute cholestasis with canalicular bile plugs, without evidence of ductopenia	1.8	ursodeoxycholic acid and cholestyramine down-trending within 60–80 days
Peranathan et al. [30]	2022	43	RAD-140				Jaundice and fatigue	Unspecified image normal	Acute cholestasis with canalicular bile plugs, without evidence of ductopenia	2.3	ursodeoxycholic acid and cholestyramine down-trending within 60–80 days
Weinblatt et al. [13]	2022	31	Enobosarm		2	0	Itch and dark-colored urine	US abdomen, fatty liver, otherwise normal		7.5	Cyproheptadine, labs significantly improved after 7 days and normalized at 10 month follow up
Wallstab et al. [31]	2022	37	Ligandrol (LGD-4033)	4 mg	8	60	Jaundice, pruritus, anorexia, fatigue, 12 kg weight loss, dark urine	U/S abdomen ruled out extrahepatic cholestasis and cholecystolithiasis Fibroscan showed fibrosis 10.7 ± 2.3 kPa	Canalicular cholestasis, ductopenia, Acute portal hepatitis with early periportal fibrosis	2.7	Ursodeoxycholic acid and cholestyramine, Linimentum Aquosum ointment

**Table 2:** Summary of clinical trials.

Author	Year	Study Design	SARM	Study Population	Study Arms and Total Subjects	Summary of Adverse Events
Marcantonio et al. [32]	2010	Randomized, double-blind, placebo-controlled, parallel group	MK-3984 and MK-2866	Healthy postmenopausal women	Placebo (N = 22) MK-2866 3 mg daily (N = 22) MK-3984 50 mg daily (N = 22) MK-3984 125 mg daily (N = 22) Total exposed = 66 Total N = 88	7/44 subjects receiving the 50 mg or 125 mg dose of MK-3984 had ALT elevation > 3 × ULN and were discontinued from the study, but no significant ALT elevation was reported for the 3 mg dose of MK-2866.
Meglasson et al. [33]	2010	Double-blind, placebo-controlled ascending single dose	LGD-4033	Healthy adult males	0.1–22 mg	No SAE’s reported.
Dalton et al. [34]	2011	Double-blind, placebo-controlled phase II trial	Enobosarm	Elderly men and postmenopausal women	Daily doses of placebo, 0.1, 0.3, 1, or 3 mg of Enobosarm for 86 days. (N = 24 per group, 50% male and female) Total Exposed = 96 Total N = 120	No SAE’s reported. ALT increased in dose-dependent fashion (8 total patients with 6 of 8 in highest dose 3 mg group). In these 7 patients, ALT elevations resolved while on drug. One subject discontinued (3 mg dose) due to ALT elevation of 4.2 X ULN and returned to normal after drug discontinuation. Dose-dependent reduction in HDL.
Yi et al. [35]	2012	Pharmacokinetic and Metabolism Study	LY2452473	Healthy males	15 mg (N = 6) Total Exposed N = 6 Total N = 6	No safety events reported.
Basaria et al. [36]	2013	Placebo-controlled ascending single and multiple dose PK	LGD-4033	Healthy adult males	Placebo (N = 33) 0.1 mg (N = 18) 0.3 mg (N = 11) 1 mg (N = 14) Total Exposed = 43 Total N = 76	No SAE’s, no study discontinuation due to adverse events, no clinically significant changes in liver enzymes. Dose-dependent reduction in HDL but returned to baseline 25 days post-last dose.
Papanicolaou et al. [21]	2013	Randomized placebo-controlled efficacy and safety	MK-0773	Females with sarcopenia	Placebo (N = 89) MK-0773 50 mg BID (N = 81) Total Exposed N = 81 Total N = 170	13.5% (N = 23) of participants had an AE possibly, probably or definitely related to study therapy. The most common AE leading to discontinuation was an increase in ALT (N = 5, 6.2%). This occurred within 6–8 weeks and all ALT returned to baseline with no clinical sequelae after stopping therapy. Hematocrit increases >3% were significantly more frequent in the MK-0773 group compared with placebo. Trend towards increased blood pressure in MK-0773 compared with placebo (5 mmgHg difference from baseline at 6 months in treatment vs. placebo group).
Bhattacharya et al. [37]	2016	Placebo-controlled ascending single and multiple dose PK	PF-06260414	Healthy adult males	Western: Placebo N = 17 SAD: 1–400 mg single dose (N = 6 per group) MAD: 3–100 mg BID (N = 6 per group) Japanese: Placebo (N = 2) 30 mg BID (N = 5) Total Exposed = 53 Total N = 72	No severe AE’s. A total of 42 of 67 treatment-emergent AE’s were considered treatment-related. Three subjects receiving SARM discontinued the study (anxiety, hypertension and ALT increase). ALT increase occurred in 10 subjects (9 SARM subjects and 1 placebo subject). Five subjects receiving 100 mg BID had ALT increases and 3 Japanese subjects receiving 30 mg BID had ALT increases. One subject in 100 mg BID cohort had ALT increases as early as day 6 with peak of 343 IU/L on day 14, which normalized by day 42 leading to discontinuation. Dose-dependent decreases in HDL
Coss et al. [38]	2016	Drug–drug interaction study, crossover design with 6 to 10-day washout period	Enobosarm	Healthy adult males	All subjects dosed with Enobosarm 3 mg. The following studies had two occasions of Enobosarm and one occasion of the additional drug: Itraconazole 200 mg (N = 12) Rifampin 600 mg (N = 12) Probenecid 500 mg (N = 15) The following two studies had one occasion of Enobosarm and two occasions of the following drugs: Celecoxib 200 mg (N = 42) Rosuvastatin 10 mg (N = 49) Total Exposed N = 132 Total N = 132	No SAE’s, 21% reported AE’s while taking Enobosarm, however only 8 subject’s AE’s were determined to be related to Enobosarm. The most common AE was headache.
Clark et al. [39]	2017	Placebo-controlled ascending single and multiple dose PK	GSK2881078	Healthy adult males and postmenopausal women	SAD: 0–0.2 mg, N = 10 all males) MAD: Placebo (N = 22) 0.05–0.75 mg (N = 67, 24 female subjects) Total Exposed N = 77 Total N = 99	Dose-dependent reductions in HDL. Female subject on active treatment developed a maculopapular rash and ALT increase to 2.9 X the ULN and discontinued the study. Another female developed and ALT elevation to 2.3 X the ULN. Two men on active treatment developed muscle soreness after demanding activity in the follow-up period 14 and 28 days, respectively, with elevation in CPK (17,841 and 4590 IU, respectively and mild elevations in ALT, both resolved over 3 weeks). No other subjects showed ALT ≥ 2 X ULN during treatment.
Neil et al. [40]	2018	Randomized double-blinded, placeb0-controlled dose-escalation PKPD	GSK2881078	Healthy elderly men and women	Cohort 1: Males: 0.75 mg or 1.5 mg Females: 0.5 mg or 0.75 mg Dosed twice daily for 3 days followed by daily for 28 days. (N = 10 each dosing group, 2:1 placebo) Cohort 2: Males: 4 mg daily for 56 days Females: 0.35 mg daily for 28 days followed by 1.5 mg daily for 28 days (N = 10 in each dosing group, 2:1 placebo) Total exposed N = 62 Total N = 93	62.9% receiving SARM compared with 13.3% placebo had ALT elevations. All elevations in ALT were transient occurring from approximately day 14 beginning to resolve and returning to baseline by day 28 of dosing. ALT incidence was most frequent in highest dose group 4 mg, with 2 individuals having a 5–10 X ULN elevation in ALT.
Peters et al. [41]	2018	Single arm, non-randomized efficacy study	GTX-024 (Enobosarm)	Postmenopausal females with stress incontinence	3 mg daily Total Exposed N = 19 Total N = 19	Minimal adverse events with none above Grade I. Elevated ALT in 1 of 19 subjects.
Ristic et al. [42]	2018	Randomized placebo-controlled study	VK5211 (ligandrol)	Patients >65 recovering from hip fracture 3–7 weeks prior	Placebo 0.5 mg daily 1 mg daily 2 mg daily Total N = 108, number in each arm not specified.	No SAE’s reported.
NCT01401543 [43]	2019	Bioavailability study crossover design	LY2452473	Healthy male	5 mg LY2452473 + 5 mg tadalafil standard and at three different particle sizes (10, 40 and 90 microns) single dose Total Exposed = 24 Total N = 24	No SAE’s, most common AE was headache, no report of liver enzymes.
NCT01160289 [44]	2019	Randomized placebo-control trial	LY2452473	Males with erectile dysfunction	5 mg tadalafil + placebo (N = 87) 10 mg tadalafil + placebo (N = 89) 1 mg LY2452473 + tadalafil daily (N = 85) 5 mg LY2452473 + tadalafil daily (N = 97) 5 mg LY2452473 + placebo (N = 52) Total Exposed N = 234 Total N = 410	SAE’s occurred slightly more commonly in patients randomized to LY2452473 (4 of 234 subjects) compared with 0 of 176 subjects randomized to a placebo group. SAE’s included humerus fracture, pulmonary embolus, tubular interstitial nephritis, arrythmia, lobar pneumonia and cardiac arrest. Association with SARM not noted. Other AE’s occurred at similar rates between SARM and placebo groups (20–30% of patients). No patient in the SARM groups had AST elevation.
NCT03241342 [45]	2020	Randomized placebo-controlled study	GTX-024	Females 18–80 years old with stress incontinence	Placebo (N = 165) GTX-024 1 mg daily (N = 163) GTX-024 3 mg daily (N = 163) Total Exposed N = 326 Total N = 491	5 total SAE’s in GTX-024 groups vs. 4 in placebo. SAE’s included hip fracture, dysesthesia, myocardial infarction, cerebral vascular accident and goiter. Non-SAE’s were also similar in GTX-024 vs. placebo group. Only 2 of 326 subjects experienced increased ALT.
Efimenko et al. [46]	2021	Cross-sectional survey study in SARM users	LGD-4033 RAD-140 MK-2866	Most commonly healthy adult men (98.5%) that were 18–29 (72.3%) years old	Total survey responders N = 343	54.5% of users reported adverse effects related to SARM use. The most common were mood swings (22.4%), decreased testes size (20.7%) and acne (15.2%). The proportion of responders reporting a side effect increased significantly with longer reported exposure times to SARM.
Pencina et al. [47]	2021	Randomized placebo-controlled, double blind	OPK-88004	Prostate cancer survivors	Placebo N = 36 1 mg daily N = 28 5 mg daily N = 14 15 mg daily N = 14 Total Exposed N = 78 Total N = 114	3 SAE’s not considered treatment-related were coronary artery bypass grafting, renal cancer and lung and liver cancer. Dose-dependent increases in AST and ALT with only one participant in the 15-mg group having AST and ALT elevations above the ULN. Dose-dependent decreases in HDL.
NCT03297398 [48]	2021	Randomized placebo control trial	OPK-88004	Males with symptoms of benign prostatic hyperplasia	Placebo (N = 38) OPK-88004 15 mg (N = 40) OPK-88004 25 mg (N = 38) Total Exposed N = 78 Total N = 114	2 subjects had SAE’s in 25 mg group (coronary artery disease, biliary obstruction and cholangitis)—association not noted. Other AE’s occurred at much higher rates in SARM groups (60–70%) compared with placebo (10–15%). This appeared to be heavily driven from lab abnormalities such as decreased testosterone, decreased LDL and decreased HDL. ALT increased in 4 subjects randomized to SARM (3/40 and 1/38 in the 15 mg and 25 mg groups, respectively).

## Data Availability

The data presented in this study are available on reasonable request from the corresponding author.

## Supplementary Materials

The following supporting information can be downloaded at: https://www.mdpi.com/article/10.3390/jox13020017/s1, PRISMA Flow diagram [75].
