# Supplementary material for: Systematic Review of Safety of Selective Androgen Receptor Modulators in Healthy Adults: Implications for Recreational Users
Source: J Xenobiot. 2023 May 10;13(2):218–36. doi: 10.3390/jox13020017 (PMC10204391; doi:10.3390/jox13020017)
Supplement: Supplementary file 1 [file jox-13-00017-s001.zip › jox-2334121-supplementary.pdf]

PRISMA 2020 flow diagram for new systematic reviews which included searches of databases and registers only.

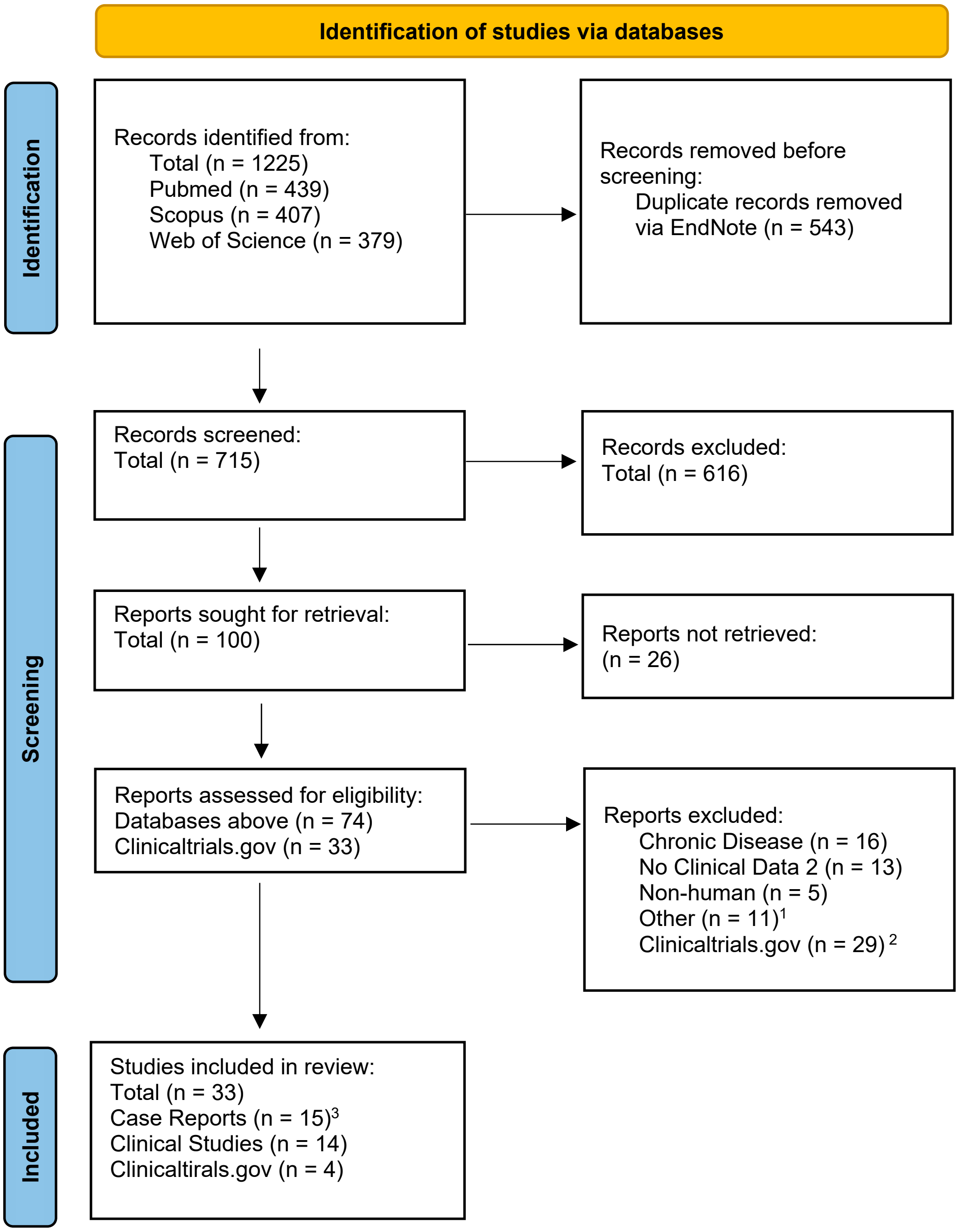

1 Reasons include not a clinical study or no exposure to SARM. One case report, Padappayil et al. reporting myocarditis associated with SARM use, was excluded due to confounding comorbidities in the patient presented. Guo et al. reported a secondary analysis of Pencina et al. and was excluded. Hilkens et al. estimated the prevalence of SARM use via survey but did not survey adverse effects. Peters et al. had duplicate results published as two separate abstract poster presentations.

2 Clinicaltrials.gov was screened sequentially after the screening and inclusion of studies from Pubmed, Scopus, and Web of Science was complete. From Clinicaltrials.gov, 13 studies in cancer populations, 4 studies with no SARM, 2 studies terminated with no results (extension studies stopped after lack of efficacy found for Gx-024 as a therapy for stress incontinence), 7 repeat studies found in the other databases, and 3 studies that met the inclusion criteria but had no results (NCT01538420, NCT01275157, and NCT03264651) were excluded.

3 Some case report studies reported multiple cases.

From: Page MJ, McKenzie JE, Bossuyt PM, Boutron I, Hoffmann TC, Mulrow CD, et al. The PRISMA 2020 statement: an updated guideline for reporting systematic reviews. BMJ 2021;372:n71. doi: 10.1136/bmj.n71
